# Connexin 43 Role in Mitochondrial Transfer and Homeostasis in the Central Nervous System

**DOI:** 10.1002/jcp.70086

**Published:** 2025-08-21

**Authors:** Anna Gervasi, Simona D'Aprile, Simona Denaro, Maria Angela Amorini, Nunzio Vicario, Rosalba Parenti

**Affiliations:** ^1^ Department of Biomedical and Biotechnological Sciences University of Catania Catania Italy

**Keywords:** gap junction, homeostasis, intercellular communication, metabolism, neurological disorder

## Abstract

Connexin 43 (Cx43) is a transmembrane protein involved in the assembly of gap junctions (GJs) and hemichannels (HCs), organized structures that allow the transferring of ions and small signaling molecules between cells and/or extracellular environment, thereby contributing to tissue homeostasis intercellular communication. Cx43 has recently been identified within the mitochondria of cells, suggesting that it may have additional functions beyond its canonical role. Most studies of mitochondrial Cx43 (mt‐Cx43) have been limited to cells of the cardiovascular system, where it appears to play a role in ATP production, calcium homeostasis, and the response to oxidative stress. However, its functions within the central nervous system (CNS) are not fully understood. Recently, it has been observed that Cx43‐forming GJs is one of the key mechanisms that cells use for the transfer of organelles, including mitochondria. Cx43‐mediated mitochondrial transfer is crucial in the CNS, supporting cellular homeostasis and neuroprotection under both physiological and pathological conditions. The dual roles of Cx43 in regulating mitochondrial function and in mediating mitochondrial transfer, raise important questions about how it coordinates these mechanisms. Herein, we reviewed recent findings on the importance of Cx43 and mt‐Cx43 in the healthy and altered CNS environment, with the aim of shedding light on its potential role in CNS homeostasis and as a therapeutic target in neurological disorder in which Cx43 plays a predominant function.

## 1 Introduction

Connexins (Cxs) are integral transmembrane proteins that enable cell‐to‐cell communication by forming channels allowing the rapid exchange of ions and small metabolites (Contreras et al. 2004; Goodenough et al. 1996). Six units of Cxs assemble into hemichannels (HCs), also known as connexons, which can be combined into homomeric HCs (composed of the same Cx isoform) or heteromeric (composed of different Cx isoforms) HCs (Simon and Goodenough 1998). Two HCs of adjacent cells join to form intercellular channels known as gap junctions (GJs), which play a pivotal role in direct cellular interactions (Nielsen et al. 2012).

Thus far, Cx isoforms have been shown to be encoded by 21 genes, differentially expressed in many cell types, tissues and organs (Oyamada et al. 2005). While some Cxs, such as Cx32 and Cx43, are expressed in a wide range of cells, others are found in specific organs and/or cell types. Even within the same tissue, Cxs expression pattern varies by cell type and developmental stage, indicating the cell‐type and stage‐specific, but tightly controlled, mechanisms for Cxs gene expression regulation (Oyamada et al. 2013). Cx43, encoded by the GJA1 gene, is the most widely expressed and the most studied GJ‐forming protein (Epifantseva and Shaw 2018). In the central nervous system (CNS), Cx43 participates in several cellular processes essential for maintaining homeostasis, including cell growth, signaling, and the migration of resident cells (Lapato and Tiwari‐Woodruff 2018). GJ coupling mediated by Cx43 occurs between developing neurons, astrocytes and microglia, where all cells utilize GJs to communicate and regulate key processes (Chew et al. 2010). Mutations in the coding sequence can lead to pathophysiological changes depending on which protein domain is affected (Laird 2014; Soares et al. 2015). Dysregulation of Cx43 has been implicated in the progression of neurodegenerative diseases by altering astrocytic functions, including changes in GJ communication and HC activity, which influence neuroinflammation, neuronal survival, and neurovascular unit integrity (Freitas‐Andrade and Naus 2016; Sánchez et al. 2020). Cx43 expression has been investigated in the pathophysiology of many neurological diseases and gliomas. In this context, its role appears controversial, as it can exhibit both tumor‐promoting and antitumour effects depending on its expression levels and tumor stage (Osswald et al. 2015; Uzu et al. 2018). Moreover, several independent studies have also found Cx43 in cellular organelles, including mitochondria (Peracchia 2020; J. Zhang et al. 2022). Recent evidence suggests that Cx43, typically localized in the plasma membrane, is also present within the mitochondria of various cell types, where it may influence metabolism and respiration (Boengler et al. 2012). Localization of Cx43 in mitochondria (mt‐Cx43) was first demonstrated in myocytes and cardiomyocytes. Boengler et al. reported for the first time the presence of Cx43 in the mitochondria of cardiomyocytes isolated from hearts of different species, demonstrating its involvement in mechanisms of protection from ischemic preconditioning (Boengler et al. 2005). Similarly, several following studies identified mt‐Cx43 in CNS‐resident cells, both on their outer and inner membranes. Azarashvili et al. suggested that mt‐Cx43 could play a neuroprotective role in different neuropathological conditions, since its downregulation is linked to mitochondrial dysfunction and to the increase of infarct volume after cerebral ischemia‐reperfusion (Azarashvili et al. 2011; Hou et al. 2016). Moreover, emerging studies have revealed that Cx43 can also be involved in mitochondria transfer from donor to recipient cells, in both healthy and pathological contexts (Borcherding and Brestoff 2023). Taken together, these data support the hypothesis that Cx43 is involved in GJ‐based intercellular communication, and suggest a critical role in the maintenance of metabolic homeostasis and mitochondrial function. This review aims to provide a comprehensive overview of Cx43 functions, with a particular emphasis on its mitochondrial roles. We also explore how its localization within mitochondria influences mitochondrial dynamics, energy production, and cellular responses to stress, thus unveiling its potential as a therapeutic target in neuroinflammation, neurodegenerative diseases, and CNS cancer.

## 2 Cx43 From Synthesis to Degradation

**Figure 1:**
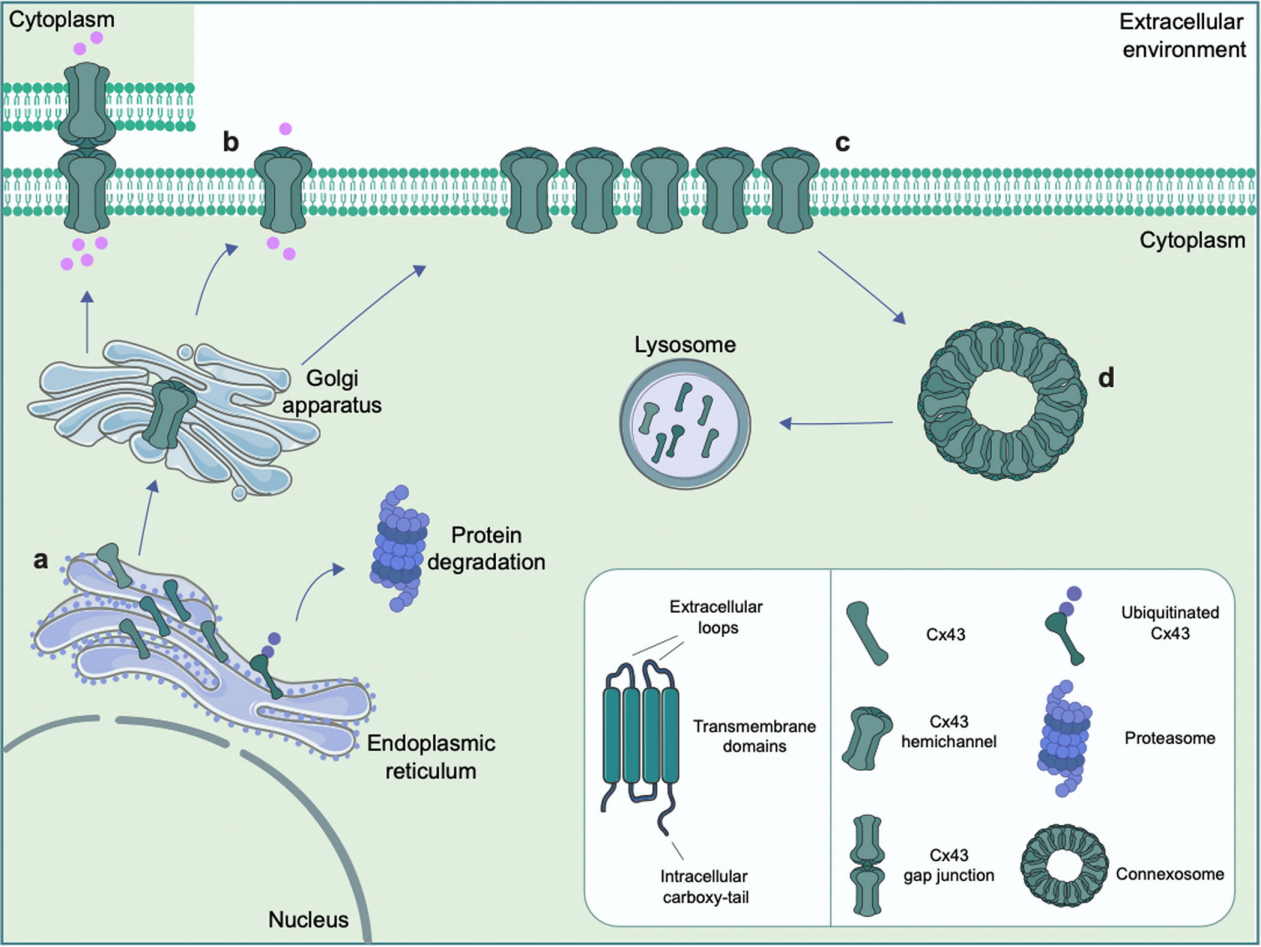
Regulation of Cx43 turnover. (a) After synthesis in the endoplasmic reticulum (ER), Cx43 oligomerizes in the Golgi apparatus and is processed in the trans‐Golgi network, before being transported to the plasma membrane. Misfolded or improperly assembled Cx43 proteins in the ER are targeted for degradation via the proteasome. (b) At the membrane, Cx43 can either form gap junctions (GJs) with hemichannels (HCs) from adjacent cells or functions as free HCs that exchange ions and small metabolites with extracellular environment. (c) GJs cluster into dynamic plaques, which undergo continuous remodeling in response to cellular signals. (d) Aged Cx43 proteins at the plasma membrane are internalized into connexosomes and degraded via lysosomal pathways.

Human Cx43 protein is encoded by the GJA1 gene and consists of four transmembrane domains, two extracellular loops (E1–E2), one intracellular loop, and N‐ and C‐terminal intracellular tails (Iyyathurai et al. 2013; Vicario and Parenti 2022). Transmembrane domains, as well as E1 and E2, are well conserved among the various Cx types, indicating their essential role in the formation of functional GJs (Hervé et al. 2007). However, the length and amino acid sequence of the intracellular loop vary substantially, contributing to the selectivity of the channels, altering not only structural conformation but also interactions with other proteins (Goodenough et al. 1996). The structural and chemical features of Cx43 have a significant impact on channel functions, such as gating dynamics and permeability. Notably, extracellular loops promote the docking of HCs from nearby cells to generate GJs. This specific structure restricts Cx43 to predominantly forming homotypic GJs, with HCs consisting entirely of Cx43 subunits. This selective pairing provides precise intercellular communication while maintaining the distinctiveness of physiological processes (Qi et al. 2023). Cx43 shows a rapid turnover with a half‐life of approximately 1.5 h and undergoes abundant posttranslational changes before oligomerizing in the Golgi apparatus, where six monomers form a HC. This oligomerization process continues in trans‐Golgi network (TGN), from which HCs move towards the plasma membrane. At the membrane, Cx43 HCs can either participate in cellular communication with the extracellular environment, or dock with HCs of adjacent cells to form GJs (Epifantseva and Shaw 2018; Hyland et al. 2021; Laird 2006). GJs can aggregate into dynamics structures called plaques, which are rapidly remodeled in response to cellular signals. Importantly, Cx43 GJs plaques undergo continuous turnover where the older subdomains are internalized by formation of annular GJ vesicles (i.e. connexosomes) and degraded by the lysosomal system (Figure 1). GJs internalization is usually a consequence of Cx43 C‐terminal tail phosphorylation which, like posttranslational modifications, is a key indicator of dynamic turnover of GJs, influencing channel gating, permeability, and interactions with cytoskeletal and regulatory proteins (Pun et al. 2022; Solan and Lampe 2014). Ubiquitination, which degrades misfolded or unassembled Cx43 proteins, also regulates Cx43 dynamics by modulating the rate of production of new Cxs in the endoplasmic reticulum (ER), as well as its degradation via the lysosomal system (Falk et al. 2016). This regulatory system is critical for maintaining adequate quantities of functional Cx43 at the plasma membrane, ensuring that GJs are correctly built and removed in response to cellular demands (Falk et al. 2016; Totland et al. 2020). Dysregulation of these pathways has been linked to a variety of disorders, including ischemic stroke, neurodegenerative diseases and cancer development (Carette et al. 2015). In the CNS, Cx43 is highly expressed on astrocytes where it is used to form stimuli‐responsive GJs, as well as to couple with other CNS‐resident cells, such as neurons, microglia and oligodendrocytes, maintaining microenvironment homeostasis (Jiang et al. 2023; Liang et al. 2020).

Given its modulatory functions on GJ assembly between astrocytes and other CNS cell populations, the phosphorylation status of Cx43 on astrocytes is an important marker in ischemic injury (Zhang et al. 2023). Evidence suggests that Cx43 phosphorylation leads to heterocellular uncoupling followed by dephosphorylation, and to GJs disassembly and increased opening of HCs (Li et al. 2015; Liang et al. 2020; Zhang et al. 2023). This phenomenon is associated with severe release of ATP and glutamate by reactive astrocytes into the extracellular milieu (Liang et al. 2020). In fact, disrupted GJs may decrease the supportive interactions between astrocytes and the other CNS cells, and an increase in HC formation may worsen neuroinflammation and brain injury (Liang et al. 2020).

Dysregulated Cx43 activity emphasizes its crucial role in neuroglial interactions, where appropriate assembly and disassembly of GJs is required to maintain CNS homeostasis. Moreover, the loss of Cx43‐based GJs in several types of tumors has been linked to enhanced cell proliferation, migration, and metastasis (Aasen et al. 2016). During tumorigenesis, aberrant Cx43 endocytosis and lysosomal degradation are frequently observed, allowing tumor cells to overcome stress conditions and increase their invasive potential (Totland et al. 2023). Aberrant degradation processes, such as altered phosphorylation and endocytic trafficking, are crucial in boosting CNS neuroinflammation and in supporting tumor invasiveness (Aasen et al. 2019). Therefore, a better understanding of Cx43 turnover and its involvement in endocytic and autophagic pathways may provide treatment prospects for these diseases.

## 3 Cx43 Intercellular Communication in CNS Pathophysiology

The Cx43 protein is differentially expressed in tissues and plays important roles ranging from regulation of neurogenesis to cell proliferation (Genet et al. 2023; McCutcheon and Spray 2022). The astrocyte network is highly interconnected by Cx43‐channels in both homeostatic and reactive conditions, allowing nonselective transfer of molecules, including ions, glucose, and signaling molecules (Boal et al. 2021; Rouach et al. 2008). Cx43‐mediated astrocyte network also supports neuronal activity. A wide range of factors modulates GJs dynamics, including neurotransmitters, neuromodulators, pro‐inflammatory molecules, and intracellular or extracellular pH and osmolarity (Giaume et al. 2021). Moreover, Cx43 GJs are involved in many CNS pathologies as indicated by significant changes in its expression and function in neuroinflammatory conditions and in many neurologic diseases (Jiang et al. 2023; Vicario et al. 2022). Indeed, the release of inflammatory mediators, which typically occurs during neuroinflammation, such as tumor necrosis factor (TNF), interleukin‐1b (IL‐1b), and interleukin‐6 (IL‐6), may impair GJ‐mediated intercellular communication (Hansson and Skiöldebrand 2015). Microglia and astrocytes communication has also been associated with the function of Cx43‐based GJs, whose dysregulation leads to either neuroprotective or neurodegenerative effects (Sarrouilhe et al. 2017; Vicario et al. 2017). Specifically, under pathological conditions such as ischemia or inflammation, the transient closure or downregulation of Cx43 GJs may act as a protective mechanism by limiting the intercellular spread of harmful signals, including excitotoxic molecules and other inflammatory mediators (Peng et al. 2022). A recent study from our group demonstrated that, in a preclinical model of neuropathic pain, the upregulation of Cx43 in astrocytes contributes to a prolonged pain hypersensitivity by enhancing cell‐to‐cell signaling, which facilitates neuroinflammation and reactive astrogliosis (Denaro et al. 2024; Vicario et al. 2022). Moreover, the reduction of heterocellular coupling between astrocytes and microglia in a preclinical model of chronic neuropathic pain mediated by a sigma‐1 receptor inhibitor, significantly ameliorates both behavioral and neuropathological signs (Denaro et al. 2024). This evidence suggests a critical role of intercellular neuroglia communication in sustaining chronicization mechanisms underlying neuroinflammation.

Cx43 also plays a key role in Alzheimer's disease (AD), where astrocytic Cx43 facilitates the propagation of toxic calcium waves and amplifies the inflammation induced by amyloid‐beta plaques, contributing to synaptic dysfunction and neuronal loss (Jiang et al. 2023; Yi et al. 2016). In physiological conditions, calcium can be released by astrocytes through Cx43‐based GJs and mediates essential functions including gliotransmission, blood flow regulation, and synaptic modulation (Murat and García‐Cáceres 2021). In pathological conditions, this mechanism becomes dysregulated with robust propagation of calcium signals across the astrocytic network. This may lead to an excessive calcium accumulation near the sites of inflammation, resulting in oxidative stress and activation of apoptotic signaling cascades in both astrocytes and the surrounding microenvironment (Giaume et al. 2019; Jiang et al. 2023). Such an increase in the resting calcium level of astrocytes has been linked with Cx43‐based HCs activity and the release of ATP and glutamate, which contribute to a further increase calcium in astrocytes (Yi et al. 2016). However, evidence from the effects of carbenoxolone and 18β‐glycyrrhetinic acid suggests that the propagation of intercellular calcium waves, evoked by inositol 1,4,5‐triphosphate (IP3) in endothelial cells, may be independent of Cx‐based channel activity (Buckley et al. 2024; Buckley et al. 2021). In addition, there are reports indicating that amyloid can activate so called “Aβ calcium channels”, which are involved in calcium release and may represent major contributors to the increase in intracellular calcium and neurotoxicity (Diaz et al. 2009).

The expression of Cxs is generally increased in AD patients CNS (Koulakoff et al. 2012). In astrocytes, this increased expression is associated with enhanced channel activity, which contributes to the propagation of inflammation and the subsequent neurotoxicity in neurons (Xing et al. 2019). Several studies have shown that targeting Cx43 activity and HCs formation, with both genetic deletion or pharmacological inhibition, reduce neuronal damage and inflammation in different AD models (Yi et al. 2016). A recent study on a mice model of AD (APP/PS1 mice) demonstrated a significant increase of Cx43 HCs activity in the hippocampus, showing that this is an early event in AD development (Madeira et al. 2023). Altered HCs activity occurs in astrocytes in the early phase of hippocampal‐dependent memory impairments and synaptic dysfunction. Such evidence is associated with cognitive and/or behavioral disorders, thus suggesting that astrocytic Cx43‐based HCs contribute to cognitive dysfunction in AD (Madeira et al. 2023). Interestingly, recent evidence demonstrates a strong association between Cx43‐based HC in microglia and cognitive deficits using a mouse model of amyloidosis (Su et al. 2025). In this study, authors showed a significant increased discrimination index in novel object recognition test in APP_swe_/PS1_dE9_ mouse model of amyloidosis with a genetic deletion of Cx43 HCs, but no effects were observed on anxiety‐like or hyperactivity behaviors (Su et al. 2025). Other in vitro studies showed that GJA1 knock‐out in astrocytes induced transcriptomic changes that overlap with gene networks implicated in AD pathogenesis (Kajiwara et al. 2018). This includes the regulation of apolipoprotein E (APOE) and other AD‐related genes, as well as pathways involved in amyloid‐beta metabolism, immune response, and synaptic function. These findings highlight the importance of this gene in AD pathogenesis and its potential as a promising pharmacological target in AD (Kajiwara et al. 2018).

Growing evidence supports a role of Cx43 in neuroinflammatory and neurodegenerative processes characterizing Parkinson's Disease (PD) (Denaro et al. 2025). Cx43 expression has been associated with microglia reactive phenotype and loss of some homeostatic functions (Faustmann et al. 2003; Froger et al. 2009). Moreover, inhibition of Cx43‐based HCs activity ameliorates neuroinflammation and degeneration of dopaminergic neurons in preclinical models of PD mediated by intranigral lipopolysaccharide injection (Zhao et al. 2022). These findings highlight the importance of Cx43 in maintaining astrocytic homeostasis and in PD pathophysiology.

Pharmacological modulation of Cxs‐based channels, particularly Cx43, has emerged as a promising therapeutic approach in neurodegenerative diseases and several compounds targeting Cx43 have shown preclinical efficacy. Gap19, a selective Cx43‐HC inhibitor, has been found to block Cx43‐HCs without impairing gap junction intercellular communication (GJIC), to prevent dopaminergic neuronal loss and to reduce microglial reactive phenotype in PD models (Lissoni et al. 2023; Maatouk et al. 2019). Another study evaluated the effect of Gap27, a Cx43‐based GJs blocker, in combination with normobaric oxygen therapy in a rat model of ischemia/reperfusion (Qi et al. 2022). This study found that the protective effects of oxygen therapy were lost by using Gap27, highlighting the importance of Cx43 (Qi et al. 2022). However, Gap27 inhibits both GJs and HCs, making it a nonselective peptide; this could block both the helpful and harmful types of the channels and this lack of selectivity may disrupt essential mechanisms of cellular homeostasis.

It is worth noticing that Cx43 is also highly expressed in many other tissues, such as cardiac and vascular tissues, where it exerts critical physiological functions (Lucaciu et al. 2023). Therapeutic strategies targeting Cx43‐based channels rise concerns on their selectivity for a specific compartment and/or tissue. As such, it is crucial to develop Cx43‐targeting strategies, selective in their mechanism of action and spatially restricted to the CNS.

As mentioned above, Cx43 GJs are involved in direct cell‐to‐cell communication in gliomas, but Cx43 exhibits a dual role acting both as a tumor suppressor and, under certain conditions, supporting tumor progression (Dong et al. 2017; Hitomi et al. 2015; Torrisi et al. 2021). Cx43 expression levels tend to decline with increasing malignancy levels. Indeed, Cx43 downregulation reduces GJIC that is crucial for maintaining cellular activity and suppressing uncontrolled proliferation (Tabernero et al. 2016). However, in the peritumoral region, functional GJs between glioma cells and astrocytes allow the transfer of microRNAs and metabolites, further supporting tumor motility and adaptability (Buruiana et al. 2020). This suggests that in the core of the tumor, low Cx43 correlates with malignancy due to reduced tumor suppressive GJIC and deregulated cell cycle control. In contrast, in the peritumoral area, high Cx43 levels in astrocytes enhance glioma cell invasiveness and contribute to the metastatic potential through direct and indirect mechanisms involving the tumor microenvironment (Aasen et al. 2016; Baklaushev et al. 2011; Sin et al. 2012). These findings highlight the crucial role of Cx43‐mediated intercellular communication in homeostatic conditions, as well as in neuroinflammation and tumorigenic environment (Table 1).

**Table 1:** Connexin 43 expression, functional role and potential therapeutic implications in neuropathic pain, Alzheimer's disease, Parkinson's disease, traumatic brain injury and glioma/glioblastoma.

Disease	Cx43 expression	Functional role	Therapeutic implications	References
Neuropathic pain	Increased in neuroglia	Promotes neuroinflammation and reactive gliosis	Opioid activation or sigma‐1 inhibition ameliorates neuropathic pain	Vicario et al. (2022)
				Denaro et al. (2024)
Alzheimer's disease	Increased in astrocytes	Propagation of inflammation and neurotoxicity	GJA1 knock‐out induces transcriptomic changes in AD‐related genes	Yi et al. (2016)
	Increased HCs activity in astrocytes and microglia of the hippocampus		Pharmacological blockade or silencing of adenosine A2A receptor prevents HCs dysregulation	Madeira et al. (2023)
			Genetic or pharmacological inhibition of Cx43‐based HCs reduces neuronal damage	Su et al. (2025)
Parkinson's disease	Increased in microglia	Promotes microglia reactive phenotype and loss of some homeostatic functions	Gap19‐mediated inhibition prevents loss of dopaminergic neurons and microgliosis	Zhao et al. (2022)
				Lissoni et al. (2023)
		Neuroinflammation and neurodegeneration	Cx43‐based HCs inhibition reverts microgliosis and neurodegeneration	Maatouk et al. (2019)
Traumatic brain injury	Unchanged total Cx43 levels	Neuroprotection	Increasing GAJ1‐20K may support neurons after damage or neurotoxic stimuli	Ren et al. (2022)
	Increased GJA1‐20K	Mitochondrial transfer from astrocytes to neurons		
Glioma/glioblastoma	Reduced in high‐malignant tumors	Tumor proliferation and tumorigenicity	Modulation of glioma/glioblastoma invasion and evaluation of the peritumoral zone	Torrisi et al. (2021)
	Increased in peritumoral astrocytes	Increased levels of Cx43 are associated with invasiveness, adhesion and migration		Tabernero et al. (2016)
		Cx43 represents a marker of peritumoral zone and reactive astrocytes associated to these areas		Baklaushev et al. (2011)

## 4 Cx43 Noncanonical Roles in CNS

**Figure 2:**
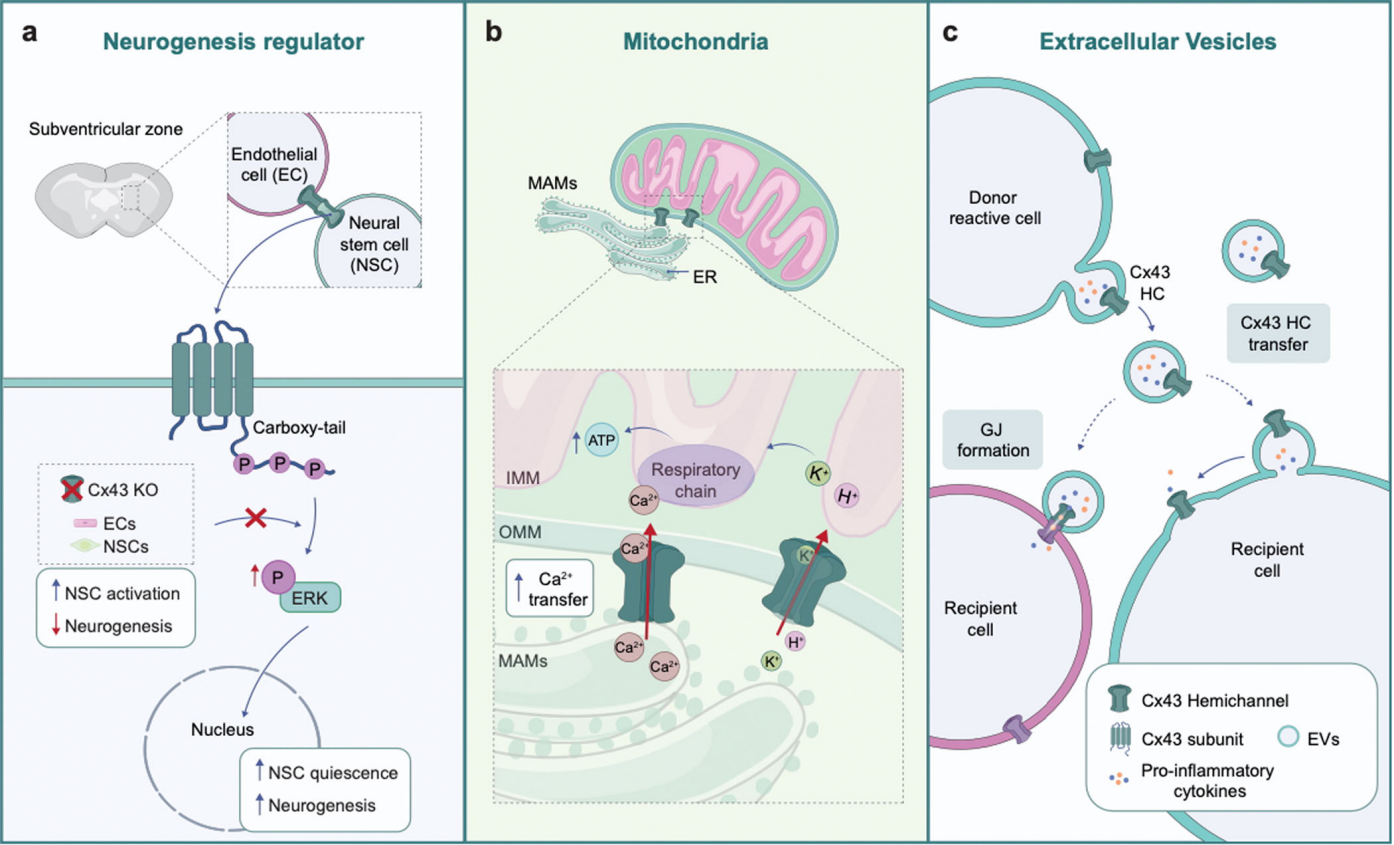
Cx43's non‐canonical roles in regulating neurogenesis, mitochondrial function and extracellular vesicles (EVs) dynamics. (a) Cx43 intracellular carboxy‐tail plays a key role in regulating the proliferation and differentiation of neuronal progenitor cells. By interacting with the with Mitogen‑activated protein kinase (MAPK) cascades and extracellular signal‐regulated kinase 1/2 (ERK) signaling pathway, Cx43 modulates neural stem cells (NSCs) quiescence, thereby fostering neurogenesis. (b) In mitochondria, Cx43 is localized on the inner mitochondrial membrane (IMM), where it governs the flux of ions, including K^+^ and H^+^. Additionally, Cx43 facilitates Ca^2+^ transfer from the mitochondrial‐associated membranes (MAMs) to the mitochondria, enhancing ATP production and supporting mitochondrial function. (c) Cx43 hemichannels (HCs) are also located on EV membrane, where they can be transferred from donor to recipient cells, impacting cellular homeostasis. This process may lead to the formation of gap junctions (GJs) between EVs and recipient cells, or fusion of EVs containing pro‐inflammatory cytokines and Cx43 HCs with recipient cells. This fusion allows recipient cells to incorporate functional Cx43 HCs, enabling further cytokines release, thereby promoting neuroinflammation.

Beyond its canonical channel function, Cx43 also plays diverse roles related to other subcellular locations, such as the nucleus, mitochondria, and extracellular vesicles (EVs, Figure 2), (Martins‐Marques et al. 2019; Xiong et al. 2024). The role of Cx43 in the nucleus has been observed in rat glioma cell line, and in a wide range of human glioma samples, suggesting Cx43 involvement in cell growth regulation (Crespin et al. 2016; Mennecier et al. 2008). Another study investigated the role of Cx43 in mediating neurovascular interactions, specifically focusing on how it influences neural stem cells (NSCs) fate in the subventricular zone (SVZ) of the adult brain (Genet et al. 2023). The authors found that Cx43, expressed by both endothelial cells (ECs) and NSCs, plays a crucial role in maintaining NSCs quiescence and in regulating neurogenesis independent of its GJ channel functions. Indeed, it has been demonstrated that Cx43‐cytoplasmic tail interacts with mitogen‑activated protein kinase (MAPK) cascades and extracellular signal‐regulated kinase 1/2 (ERK) signaling pathways to regulate NSCs' fate (Genet et al. 2023). When ERK pathway is inhibited in Cx43‐deficient ECs‐NSCs co‐cultures, the maintenance of NSCs quiescence is disrupted, confirming that Cx43 channel‐independent functions are critical in this regulatory mechanism (Figure 2a) (Genet et al. 2023). More broadly, it is known that Cx43, through its cytoplasmic tail and specifically with the cytosolic C‐terminus domain, interacts with proteins involved in many other functions (Leithe et al. 2018). Notably, it has been shown that deletion of this domain alters neuronal migration in the neocortex (Cina et al. 2009). Cina et al. have employed mice with selective Cx43 knockout in radial glial progenitors to study cellular migration, demonstrating that Cx43 expression in radial glial progenitors is essential for physiological neocortical development. In fact, electrophoretic delivery of full‐length Cx43 into knock‐out embryos can restore normal migration properties of neurons. By contrast, a C‐terminal‐truncated Cx43 protein does not rescue migration, indicating the cytoplasmic tail is essential for radial neuronal migration during neocortical development (Cina et al. 2009). Recent studies have also shown that Cx43 is localized in mitochondria, particularly in cardiac cells, where it contributes to mitochondrial calcium and potassium homeostasis (Figure 2b) (Boengler et al. 2013; Gadicherla et al. 2017). Cx43 in EVs underlies its involvement in distant cellular information exchange; indeed, a recent study suggested a potential role of Cx43 HCs in EVs as mediators of neuroinflammation (Memo et al. 2024). Particularly, it was observed in organotypic models that EVs carry functional Cx43 HCs at the membrane levels and, reactive astrocytes and microglia release EVs containing Cx43 HCs in response to inflammatory stimuli (Memo et al. 2024). Moreover, the use of GAP27, a peptide that specifically blocks Cx43 HCs, fully prevented the pro‐inflammatory response (Memo et al. 2024). Still, the exact mechanism by which Cx43‐EVs support neuroinflammation is not fully understood. It is possible that Cx43 HCs on the surface of EVs form channels with astrocytes, allowing the release of pro‐inflammatory molecules to recipient cells (Soares et al. 2015). Alternatively, EVs might directly fuse with the astrocytic membrane and insert functional Cx43 HCs promoting the release of pro‐inflammatory mediators (Figure 2c). In conclusion, emerging research reveals Cx43 noncanonical roles beyond its traditional function in GJIC. Cx43 localization in various intracellular compartments, including nucleus, mitochondria, and extracellular vesicles, highlights its potential to impact on several cellular functions in a GJ‐independent manner. Studies have demonstrated Cx43 critical involvement in regulating cell growth, neurogenesis, neural migration and stem cell quiescence through mechanisms dependent on its cytoplasmic tail and interactions with intracellular signaling pathways. Evidence suggests that Cx43 functions as a regulator of diverse cellular processes, emphasizing the need for future research to elucidate these non‐channel‐dependent roles.

## 5 Mt‐Cx43

Cx43 also localizes to mitochondria, emerging as a novel key regulator of metabolic homeostasis and mitochondrial function. Pioneering evidence pointing towards a mitochondrial localization of Cx43 were first published in 2002 (Li et al. 2002). Using immunolocalization and cell fractionation analysis, Cx43 was identified in mitochondria‐enriched fractions. The presence of Cx43 within mitochondria on cardiomyocytes was fully established using complementary experimental approaches to prove its subcellular localization, combining mitochondrial fractionation, fluorescence‐activated cell sorting, confocal‐assisted imaging and immuno‐electron microscopy (Boengler et al. 2005; Boengler et al. 2022). Subsequently Cx43 has been implicated in different aspects of mitochondrial function including fusion and fission processes, ATP generation, energy mobilization, and protection against oxidative stress (Boengler et al. 2022; Xiong et al. 2024; Zhang et al. 2022). In addition, Cx43 promotes calcium exchange between the ER and mitochondria, crucially influencing mitochondrial metabolism and energy generation (Zhang et al. 2022) (Figure 2b). In cardiac cells, mt‐Cx43 regulates potassium uptake, impacting oxygen consumption (Boengler et al. 2013; Gadicherla et al. 2017). Notably, following ischemia‐reperfusion injury, Cx43 HCs have been shown to mediate mitochondrial calcium influx, which contributes to the induction of mitochondrial permeability transition and subsequent cardiomyocyte death. In fact, inhibition of HCs using channel blockers significantly reduces calcium entry in cardiac subsarcolemmal mitochondria, thereby reducing both necrotic damage and infarct size (Gadicherla et al. 2017). Evidence suggests that Cx43 is imported into mitochondria via the TOM/TIM complex, with a mechanism that appears to be regulated by Cx43 phosphorylation (Rodriguez‐Sinovas et al. 2006). However, the mechanisms governing its import and degradation remain incomplete and need to be further addressed.

**Figure 3:**
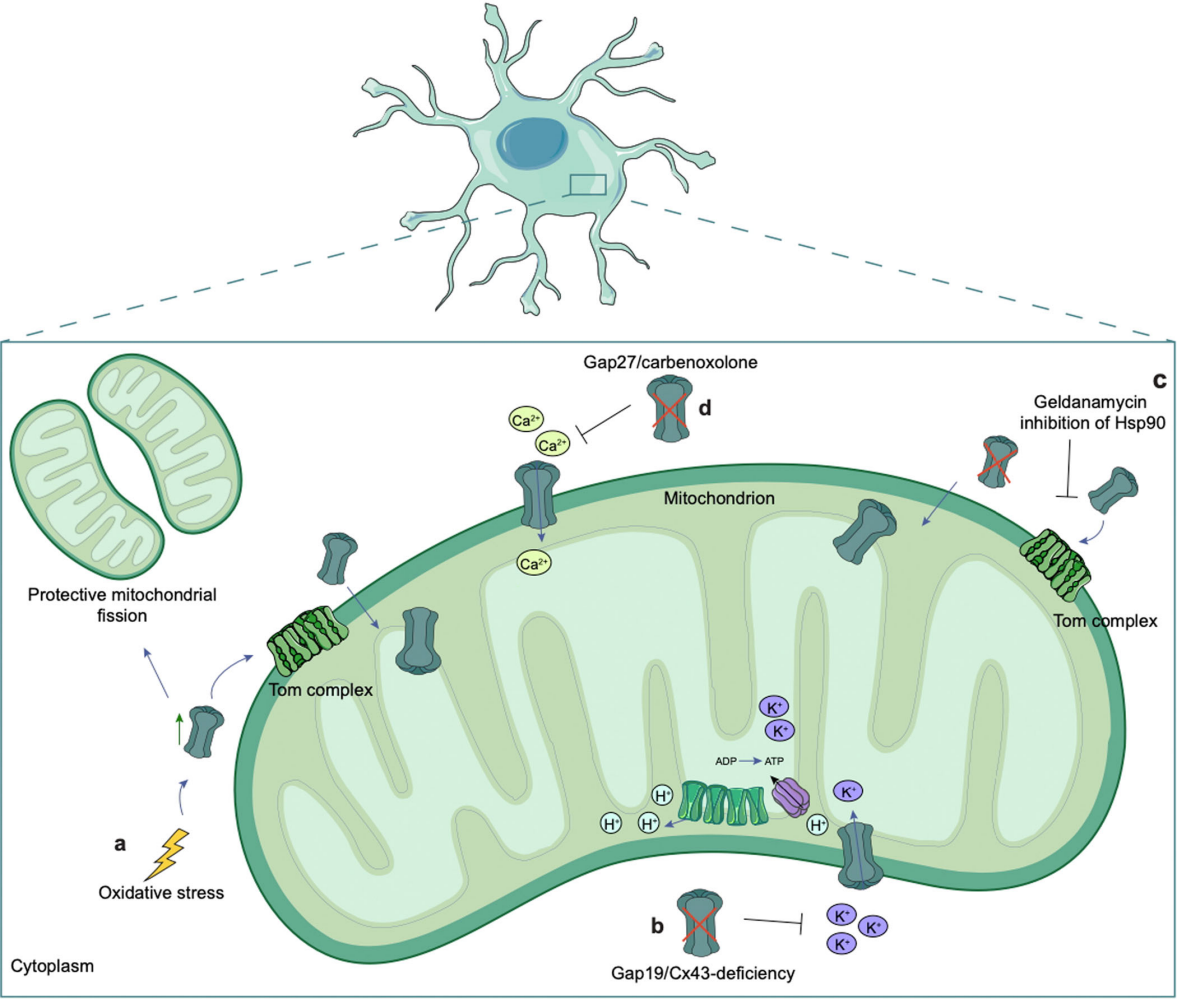
Role of mitochondrial Cx43. (a) Mitochondrial Cx43 (mt‐Cx43) plays a crucial role in mitochondrial function among which the response to oxidative stress; mt‐Cx43 expression increases and modulates mitochondrial dynamics, supporting protective mitochondrial fission. (b) mt‐Cx43 also regulates ATP production by facilitating the exchange of ions such as potassium (K^+^), critical for maintaining mitochondrial membrane potential and energy metabolism. Gap19 inhibition of Cx43 significantly reduced the entrance of K^+^ ions. (c) The import of Cx43 into mitochondria is regulated by TOM complex and HSP90 inhibition with Geldanamycin has been shown to impair this process, leading to a reduction in mt‐Cx43 levels and affecting mitochondrial function. (d) Calcium (Ca^2+^) ions flux is also regulated by mt‐Cx43, which interacts with mitochondrial‐associated membranes (MAMs) to mediate exchange of Ca^2+^ between ER and mitochondria; use of Gap27, a Cx43 hemichannel blocker, has been shown to decrease Ca²⁺ entry into mitochondria, further emphasizing the role of mt‐Cx43 in maintaining Ca²⁺ homeostasis.

Boengler et al. not only demonstrated Cx43 localization in mitochondria isolated from animal and human hearts, but they also correlate the expression and content of mt‐Cx43 with ischemic preconditioning since different cycles of ischemia/reperfusion increased mt‐Cx43 content (Boengler et al. 2005). Azarashvili et al. hypothesized mitochondria localization of Cx43 also in brain and liver. This study showed that the GJs blocker carbenoxolone (Cbx) induces the opening of permeability transition pores (PTPs) in both synaptic and non‐synaptic mitochondria. The authors suggested the PTP control is related to the phosphorylation state of Cx43 particularly at serine 368 during calcium stress and further decreased by Cbx. This study highlighted the protective function of mt‐Cx43 in maintaining calcium retention and mitochondrial membrane potential, especially in synaptic mitochondria, by preventing phosphorylation or inhibiting Cx43. Accordingly, Cx43 protects against calcium‐induced mitochondrial dysfunction and subsequent cell death by acting as a gatekeeper in mitochondrial signaling (Azarashvili et al. 2011). Further studies found that Cx43 is also expressed in the mitochondria of CNS resident cells. Using astrocyte cell cultures, Kozoriz et al. have investigated mitochondrial sequestration of potassium under pathological conditions, such as ischemia or seizures, in which elevated extracellular potassium levels were observed (Kozoriz et al. 2010). They demonstrated that mt‐Cx43 significantly contributes to potassium uptake in astrocytes' mitochondria. Using Cx43 knock‐out astrocytes or pharmacological blockers of GJs, they observed reduced mitochondrial potassium uptake compared with wild‐type cells. The authors employed potassium binding fluorescent indicators (PBFI) and membrane potential disruptors to show that mitochondrial potassium influx depends on functional Cx43 (Kozoriz et al. 2010). These findings underline that mt‐Cx43 is a pivotal mediator of ionic buffering, protecting astrocytes from excitotoxicity by limiting cytoplasmatic ions overload during excessive neuronal activity (Kozoriz et al. 2010). Using a middle cerebral artery occlusion (MCAO) model, Shuai Hou et al. investigated the regulation of mt‐Cx43 and its protective role against cerebral ischemia‐reperfusion injury. A significant reduction of mt‐Cx43 and its phosphorylated form, after cerebral ischemia‐reperfusion damage, has been demonstrated (Hou et al. 2016). In fact, GJ inhibition with Cbx reduces the damage, indicating that mt‐Cx43 could assemble as GJs. Treatment with diazoxide (DZX), an agonist of the mitochondrial ATP‐sensitive potassium (mitoKATP) channel, increases the phosphorylation ratio and restores mt‐Cx43 and phospho‐mt‐Cx43 levels, indicating a role for mitoKATP activation in stabilizing mt‐Cx43 function (Hou et al. 2016). In contrast, 5‐hydroxydecanoic acid (5‐HD), a mitoKATP antagonist, reverses the effects of DZX, resulting in greater downregulation of mt‐Cx43 and increasing mitochondrial damage. It has been demonstrated that mt‐Cx43 phosphorylation is mediated by protein kinase C (PKC); indeed, the treatment with phorbol‐12‐myristate‐13‐acetate (PMA), a PKC agonist, increased levels of phosphorylated mt‐Cx43 and reversed the negative effects of 5‐HD. In contrast, PKC inhibition via Ro‐31‐8425 significantly reduced mt‐Cx43 phosphorylation, indicating the critical role of PKC in regulating mt‐Cx43 activity (Hou et al. 2016). Functionally, mt‐Cx43 appears to maintain mitochondrial integrity during ischemia‐reperfusion injury, as evidenced by its ability to preserve mitochondrial structure, to reduce apoptosis rates, and to mitigate oxidative stress markers. Active mt‐Cx43 stabilizes mitochondrial membranes and inhibits the mitochondrial permeability transition pore (MPTP) formation, protecting cells from death. These findings suggest a dual role of mt‐Cx43 as a structural and regulatory protein in the mitochondria. By interacting with mitoKATP channels and PKC signaling, mt‐Cx43 contributes to neuroprotection in cerebral ischemia‐reperfusion injury (Hou et al. 2016). Although these studies have demonstrated the presence of mt‐Cx43 in different cell types, its specific roles within the CNS remain poorly understood. This gap in knowledge highlights the need for a deeper investigation into the potential functions and mechanisms involving Cx43 in mitochondria, which could provide valuable insights into their contribution to neural processes. The main known functions of mt‐Cx43 are summarized in Figure 3.

## 6 Mechanisms of Mitochondrial Transfer: The Role of Cx43

**Figure 4:**
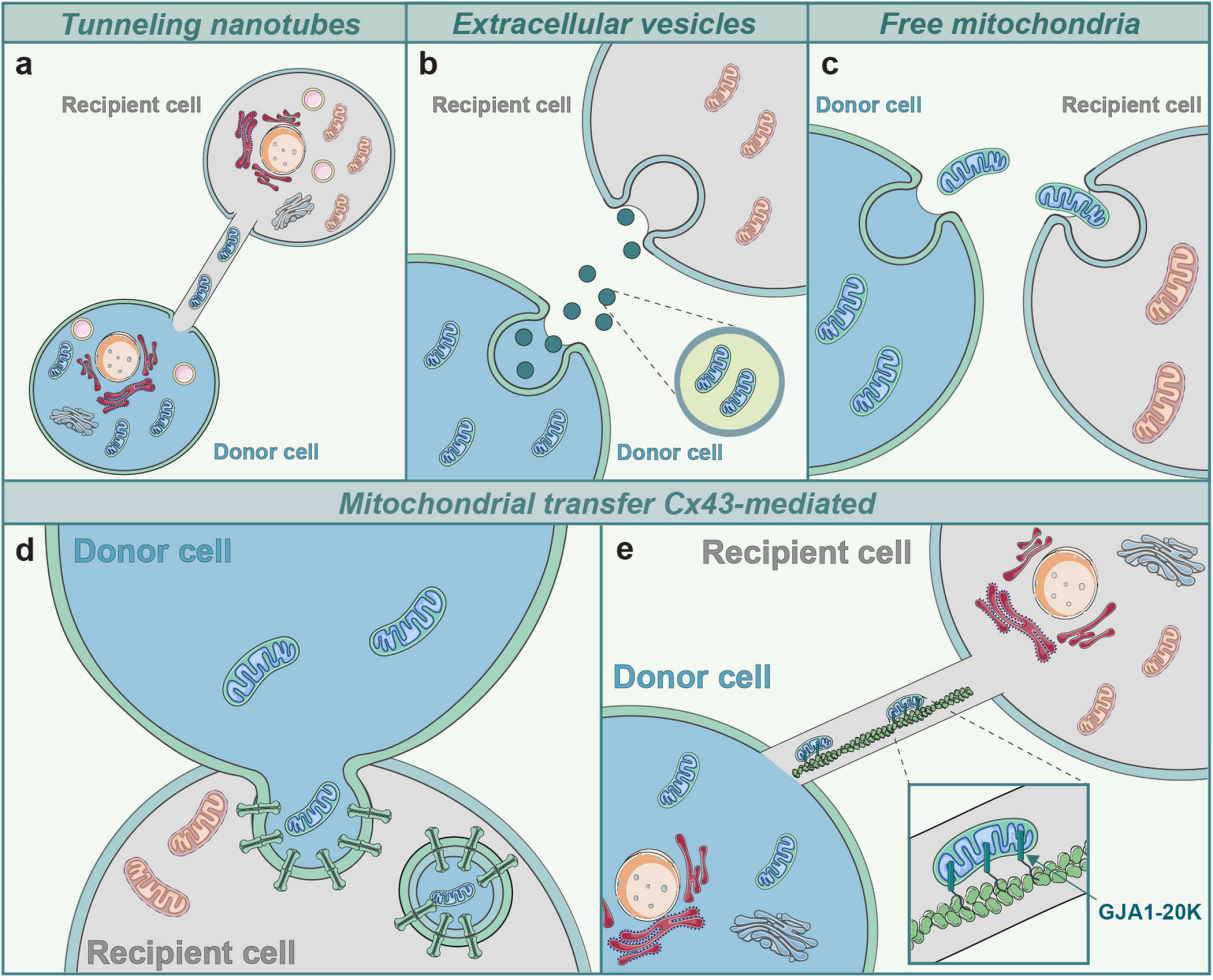
Different mechanisms of mitochondrial transfer. (a) Healthy donor cells can transfer their mitochondria through tunneling nanotubes (TNTs) to recipient cells, that possess impaired mitochondria. (b) Donor cells secrete extracellular vesicles (EVs) containing mitochondria that are captured by recipient cells through endocytosis. (c) Donor cells can also release free mitochondria in the extracellular environment, where recipient cells engulf them through a phagocytic mechanism. (d) Mitochondrial transfer can be Connexin 43 (Cx43)‐mediated, with donor cells that move mitochondria towards recipient cells through Cx43‐based GJs. (e) A small Cx43 isoform, GJA1‐20K, is involved in promoting mitochondria transfer between cells via TNTs.

Several independent studies have shown the ability of “donor” cells to transfer mitochondria to “acceptor” cells (Baldwin et al. 2024; Chen and Chen 2023; Liu et al. 2021; Liu et al. 2022). Mitochondrial transfer has attracted great interest since the passage of healthy mitochondria into damaged cells could support their metabolism. Therefore, several mechanisms underlying the intercellular transfer of mitochondria have been proposed. One main mechanism involves the formation of a direct connection between cells through tunneling nanotubes (TNTs) (Figure 4a) (Borcherding and Brestoff 2023; Irwin et al. 2024; Norris 2021).

Another mechanism proposed the release of extracellular vesicles (EVs) containing mitochondria to be captured by recipient cells (Figure 4b). This mechanism has been associated with nicotinamide adenine dinucleotide (NAD^+^)/CD38/cyclic ADP ribose (cADPR)/calcium pathway activation, in which accumulating calcium concentrations promote actin remodeling and the subsequent cell membrane invagination, leading to EVs endocytosis (Qin et al. 2021).

Finally, the release of free mitochondria into the extracellular environment has also been described; indeed, they are captured by acceptor cells through a phagocytic mechanism (Figure 4c, (Borcherding and Brestoff 2023). Several studies have shown mitochondrial transfer in the CNS and their therapeutic potential (Hayakawa et al. 2016; Peruzzotti‐Jametti et al. 2021). An in vivo study using a transient focal cerebral ischemia mice model demonstrated that astrocytes transfer mitochondria to adjacent neurons. This process involves a mechanism mediated by CD38 and cyclic ADP‐ribose, facilitating the release of mitochondria through EVs. Suppression of CD38 signaling reduces neuronal recovery and decreases mitochondrial transfer (Hayakawa et al. 2016). Moreover, it has been observed that microglia exposed to α‐synuclein can transfer accumulated protein from donor to acceptor cells through TNTs (Scheiblich et al. 2021). Homeostatic microglia receive α‐synuclein and donate mitochondria to cells overloaded with α‐synuclein, reducing reactive oxygen species (ROS) levels and supporting the survival of donor cells exposed to cytotoxic stress mediated by α‐synuclein (Scheiblich et al. 2021).

Mitochondrial exchange between cells has been linked to Cx43‐mediated GJ formation (Figure 4d). Formation of TNTs often depends on Cx43, and silencing Cx43 reduces mitochondrial transfer between cells (Tishchenko et al. 2020). Cx43‐mediated mitochondrial transfer has also been observed in the CNS, both in physiological and pathological conditions. Particularly, Norris demonstrated, by electron microscopy and immunogold labeling, that whole mitochondria and endosomes can be incorporated into double‐membrane connexosomes, which facilitate mitochondrial transfer (Norris 2021).

Recent studies explored the role of Cx43 in mediating mitochondrial transfer from astrocytes to neurons in case of traumatic brain injury (TBI). Authors investigated the role of Cx43 small isoform (i.e. GJA1‐20K) in TBI demonstrating that upregulated Cx43 expression in astrocytes promotes mitochondrial transfer from astrocytes to neurons and neuroprotection after TBI‐like damage in vitro (Figure 4e) (Ren et al. 2022). Through MitoTracker, astrocytes mitochondria have been labeled to track mitochondrial transfer from astrocytes to neurons, demonstrating the presence of astrocytes mitochondria within neurons. In addition, inhibiting Cx43 HCs with Gap26, mitochondrial transfer was almost completely blocked indicating that GJA1‐mediated mitochondrial transport is an essential process in the repair of damaged neurons (Ren et al. 2022). It is worth noticing that, whether in vivo preclinical studies on mitochondrial transplantation in TBI reported a significant amelioration of cognitive and/or behavioral outcomes (Bamshad et al. 2023; Zhang et al. 2020), to the best of our knowledge, no study has linked in vivo transplantation of mitochondria and Cx43‐based transfer in TBI models.

While it is well accepted that mitochondrial transfer mediated by Cx43 is a physiological phenomenon to maintain homeostasis in CNS cells, it remains unclear if this mechanism is also present in other pathological conditions, such as tumors. Indeed, recent studies have demonstrated mitochondrial transfer in GBM cells through tumor microtubes (TMs) that show a structure similar to TNTs, identifying them as mediators of mitochondria exchange between malignant cells (da Silva et al. 2019; Osswald et al. 2015). The same mechanism is used by GBM cells to acquire functional mitochondria from nonmalignant cells, such as astrocytes, to increase their metabolic activity and tumorigenicity (Watson et al. 2023). However, none of these exchanging mechanisms in GBM tumor microenvironment (TME) have been linked yet to Cx43, even though studies have revealed the presence of Cx43 at the TMs, suggesting a role of Cx43 GJs‐mediated communication in TMs stabilization (Osswald et al. 2015; Weil et al. 2017). The presence of Cx43 in TMs and the mitochondrial transfer between GBM and CNS cells deserves further investigations to demonstrate that mitochondrial transfer might be mediated or regulated by Cx43 in GBM, as observed in other CNS cells.

## 7 Therapeutic Potential of mt‐Cx43 in CNS Disorders

In neurodegenerative disorders, ROS overproduction leads to oxidative stress (Singh et al. 2019). Similarly, in GBM, cellular redox mechanisms are highly impaired, and ROS production is correlated with tumor progression and drug resistance (Singh et al. 2019). Modulation of mt‐Cx43 expression could enhance recovery from cellular damage and reduce oxidative stress and, therefore, it could be a potential target for CNS‐related pathologies by exerting neuroprotective and anti‐inflammatory effects. In GBM, mt‐Cx43 could also influence tumor cells metabolism, representing a strategy to reduce therapy resistance. Current treatments for GBM include surgical resection, radiotherapy, and chemotherapy with temozolomide (TMZ) (Jezierzański et al. 2024). However, standard therapy is unsuccessful in the majority of GBM cases due to cellular heterogeneity, resistance to therapy, and the infiltrative nature of tumor cells, which hinders a complete surgical resection (Osswald et al. 2015).

Since metabolism and mitochondrial dynamics have been identified as GBM hallmarks, mt‐Cx43 could play a significant role in shaping its phenotype (Lu and Ho 2020). Targeting mt‐Cx43 could may reduce tumor metabolism and oxidative stress, disrupting key metabolic pathways and limiting cell proliferation. However, further research is needed to fully elucidate the role of mt‐Cx43 in GBM and to develop targeted therapies that can effectively exploit its vulnerabilities. More effective therapies could be those that exclusively target mt‐Cx43, preserving the general physiological functions of Cx43 in normal tissues, such as intercellular communication. ROS play a considerable role in the progression of neurodegenerative diseases leading to oxidative stress that affects cellular structures, mitochondrial activities, and inducing neuronal death (Zhou et al. 2022). Therefore, treating mitochondrial dysfunction, which is a common feature in neurodegenerative diseases, could be a potential strategy against these disorders (Rehman et al. 2023). In this regard, mt‐Cx43 might be a good candidate due to its role in the maintenance of mitochondrial balance, ROS synthesis and responses to cellular stress. Mt‐Cx43 also participates in the modulation of MPTP dynamics, which are crucial for maintaining mitochondrial membrane potential, calcium balance and ROS generation (Ferko et al. 2019). Abnormal opening of MPTP, which is a characteristic of neurodegenerative diseases, worsens oxidative injury, inducing apoptotic events (Bonora et al. 2020). It might be possible to prevent ROS overproduction by reestablishing the MPTP mechanisms through targeted modulation of mt‐Cx43. In the case of AD, damaged mitochondria accumulate due to abnormal mitophagy and excessive ROS production. An assessment of the enhanced mt‐Cx43 expression in AD models and its functions would shade lights on its role in maintaining mitochondrial integrity and regulating excessive ROS levels.

## 8 Conclusions

Recent studies have revealed that Cx43 is much more than a channel protein; it is a versatile protein involved in different processes, such as CNS development, cellular repair, and even disease progression. The rapid Cx43 turnover underlines its highly dynamic and tightly regulated nature, which enables the protein to respond to the cellular environment. This highly regulated system guarantees that its expression levels are closely monitored, since even slight alterations can result in significant poor outcomes. Cx43 is also modified post‐translationally, and these modified sequences and structures influence its stability, position and functional properties. Subcellular Cx43 localization shifts from the nucleus to the membrane, and even to the mitochondria. This phenomenon further underscores the fundamental role of this protein, particularly in the CNS physiology, where Cx43 is the most highly expressed. In mitochondria, Cx43 seems to be involved in energy production, regulates calcium levels, and protects cells from stress effects. Moreover, mitochondrial transfer mediated by Cx43 is crucial to maintain homeostasis and to provide metabolic support. For instance, astrocytes mediate the processes of neuronal recovery after injury thanks to the transfer of healthy mitochondria. The question is, how can Cx43 be present inside mitochondrial membranes, performing HCs activity, and at the same time be a mediator of mitochondrial transferring between cells? The different roles of Cx43 in mitochondria, one as a protein related to mitochondrial transfer and another as a protein that is involved in mitochondrial homeostasis, needs to be addressed. The identification of Cx43 in mitochondria opens new opportunities for further research on other Cx43 functions beyond its well‐known tasks. Given its potential therapeutic implications, further investigation is needed to explore its role in GBM and in neurodegenerative diseases. Understanding the mechanisms involved in mt‐Cx43's functions might help in finding new targets for therapeutic approaches to reduce disease progression and to improve patient outcomes. The insufficient number of studies on this topic underlines the critical requirement for further research to gain more insights on the different mt‐Cx43 functions.

## Author Contributions

The study was conceptualized by Anna Gervasi, Nunzio Vicario, and Rosalba Parenti. The original draft was prepared by all authors, with all authors also contributing to the review and editing process. Visualization was carried out by Anna Gervasi, Simona D'Aprile, and Simona Denaro, while supervision was provided by Nunzio Vicario and Rosalba Parenti.

## Conflicts of Interest

The authors declare that the research was conducted in the absence of any commercial or financial relationships that could be construed as a potential conflicts of interest.

## Data Availability

Data sharing is not applicable to this article, as no new data were created or analyzed in this study.
